# Isolation of Alpha-Glucosidase Inhibitors from the Panamanian Mangrove Plant *Mora oleifera* (Triana ex Hemsl.) Ducke

**DOI:** 10.3390/ph17070890

**Published:** 2024-07-04

**Authors:** Lilia Cherigo, Javier Liao-Luo, Juan Fernández, Sergio Martínez-Luis

**Affiliations:** 1 Departamento de Química Orgánica, Facultad de Ciencias Naturales, Exactas y Tecnología, Universidad de Panamá, Ciudad de Panamá P.O. Box 3366, Panama; lilia.cherigo@up.ac.pa (L.C.); javierliao98@gmail.com (J.L.-L.); 2 Centro de Biodiversidad y Descubrimiento de Drogas, Instituto de Investigaciones Científicas y Servicios de Alta Tecnología (INDICASAT AIP), Edificio 208, Ciudad del Saber, Apartado 0843-01103, Panama; juanor_fer11@hotmail.com

**Keywords:** *M. oleifera*, α-glucosidase inhibition, pentacyclic triterpenes, GC-MS, GNPS

## Abstract

Panama boasts an expansive mangrove area and stands as one of the most biodiverse countries in America. While mangrove plants have long been utilized in traditional medicine, there are still unstudied species whose potential medicinal applications remain unknown. This study aimed to extract bioactive compounds from *Mora oleifera* (Triana ex Hemsl.) Ducke, an understudied mangrove species. Through bioassay-guided fractionation of the crude extract, we isolated seven active compounds identified as lupenone (**1**), lupeol (**2**), α-amyrin (**3**), β-amyrin (**4**), palmitic acid (**5**), sitosterol (**6**), and stigmasterol (**7**). Compound structures were determined using spectroscopic analyses, including APCI-HR-MS and NMR. Compounds **1–7** displayed concentration-dependent inhibition of the alpha-glucosidase enzyme, with IC_50_ values of 0.72, 1.05, 2.13, 1.22, 240.20, 18.70, and 163.10 µM, respectively. Their inhibitory activity surpassed acarbose, the positive control (IC_50_ 241.6 µM). Kinetic analysis revealed that all compounds acted as competitive inhibitors. Docking analysis predicted that all triterpenes bonded to the same site as acarbose in human intestinal alpha-glucosidase (PDB: 3TOP). A complementary metabolomic analysis of *M. oleifera* active fractions revealed the presence of 64 compounds, shedding new light on the plant’s chemical composition. These findings suggest that *M. oleifera* holds promise as a valuable botanical source for developing compounds for managing blood sugar levels in individuals with diabetes.

## 1. Introduction

Plant-derived compounds have been identified as effective alpha-glucosidase inhibitors, crucial in modulating postprandial hyperglycemia. Many plant families, including mangrove plants, have been investigated for bioactive compounds with potential alpha-glucosidase inhibitory activity. Some of these plant families include Fabaceae, Zingiberaceae, Moraceae, Asteraceae, Taxaceae, Rutaceae, Lamiaceae, Combretaceae, Clusiaceae, Celastraceae, and Cucurbitaceae, among others [1,2,3].

Several plant molecules exhibit potent alpha-glucosidase inhibitory activity and are considered promising drug candidates for treating type 2 diabetes. Plant compounds like Taxumariene F, Akebonoic acid, Morusin, Rhaponticin, Procyanidin A2, Alaternin, Mulberrofuran K, and Psoralidin show potential for managing hyperglycemia by targeting alpha-glucosidase enzymes [2].

Studies aimed at revealing the bioactivity potential of mangrove plants have yielded promising results. These studies have not only uncovered the mangrove’s capacity to produce alpha-glucosidase inhibitors but have also highlighted their broader potential as a source of a diverse array of bioactive natural compounds, including steroids, triterpenes, saponins, flavonoids, alkaloids, and tannins. The rich chemical diversity of mangrove plants makes them valuable drug discovery and development resources. Moreover, the traditional use of these plants in various folklore medicine systems further indicates their potential medicinal properties and therapeutic applications [4,5].

Panama’s extensive coastline and favorable environmental conditions have led to a megadiverse mangrove ecosystem. The country offers an ideal setting for scientific exploration due to its abundant plant and animal species, which have led it to be considered a biodiversity hotspot. Panama has one of the richest mangrove populations in the Americas, hosting 11 of the 12 mangrove species documented in the entire continent, according to the World Atlas of Mangroves [6,7].

Regrettably, over the past 50 years, Panama has lost more than half of its original mangrove areas. Some reports have indicated that by 1969, there were more than 360,000 hectares of mangrove zones in the country; that number had dropped to just 170,000 hectares in 2007 [6]. These data point out that there exists a big concern related to the fact that people do not fully appreciate the value of mangroves, and on the other hand, it is evident that the survival of the different local mangrove species is highly threatened, and therefore, actions are needed to revalue this ecosystem [8].

One of the endangered mangrove plant species in Panama is *Mora oleifera* (Triana ex Hemsl.) Ducke (Fabaceae). This species is highlighted in a vulnerable condition according to the International Union for Conservation of Nature Red List of Threatened Species (IUCN) [9]. *M. oleifera* is a tall tree that grows between 15 and 45 m high. It has shallow roots that spread out over a long distance. This mangrove species produces large seeds, among the largest in the plant kingdom. The wood from *M. oleifera* trees is of high quality and is often used as a building material by the local population in the nearby areas. The tree of *M. oleifera* also produces a dye that has practical uses [9,10]. Interestingly, there are no known traditional medicinal uses for this plant species. However, this does not necessarily mean that this plant has no medicinal properties beneficial to humans, since there are no reports about the ethnomedical uses, let alone on the secondary metabolites that produce this plant.

Considering the threats faced by mangrove plants globally, the fact that *M. oleifera* is an endemic mangrove plant found along Colombia, Costa Rica, and Ecuador, and the lack of scientific knowledge regarding the medicinal properties of this mangrove plant, our research group established a systematic bioprospecting study to identify the medicinal properties this plant might have. In an initial step, we evaluated the organic extract of different mangrove species collected from the Panamanian Pacific, and we found that the organic extract from *M. oleifera* showed significant inhibition of the enzyme alpha-glucosidase, which is a well-known anti-diabetic target [11]. Therefore, the main goal of the present work was to conduct a detailed chemical analysis to identify the potentially active compounds produced by *M. oleifera*.

## 2. Results and Discussion

### 2.1. Activity-Guided Isolation and Identification

*Mora oleifera* leaves were collected from the protected mangrove area of Pedregal Port, situated in Chiriquí province, Panama. Initially, a small-scale extract was prepared using 100 g of dried leaves of *M. oleifera* by employing a mixture of dichloromethane and methanol in a 1:1 ratio. The obtained crude extract was then tested to identify its possible antiparasitic, anticancer, and antibacterial activities. In addition, the effect of the extract in the lethality test against *Artemia salina* and on alpha-glucosidase enzyme was analyzed. During this preliminary assessment, it was observed that the organic extract of *M. oleifera* only exhibited significant activity solely against the alpha-glucosidase enzyme, with an inhibition rate of 78.0% at a concentration of 6.25 mg/mL. Consequently, this last assay was chosen to guide the fractionation process of the *M. oleifera* organic extract.

A large-scale extract was prepared to initiate the fractionation process to identify the compounds active against the alpha-glucosidase enzyme contained in the organic extract of *M. oleifera*. For the initial fractionation, a sequential extraction by maceration was performed using three solvents of increasing polarity (hexane, dichloromethane, and methanol) using 890 g of dried *M. oleifera* leaves. As part of the bioguided approach, the three initial fractions were evaluated, revealing that the hexane fraction exhibited the highest activity (85.0% of inhibition), followed by the dichloromethane fraction (51.4% of inhibition). In contrast, the methanol fraction showed the lowest activity (26.7% of inhibition). These results also showed us that the main active compounds were found in the hexane fraction, so this fraction was selected for further fractionations.

**Figure 1:**
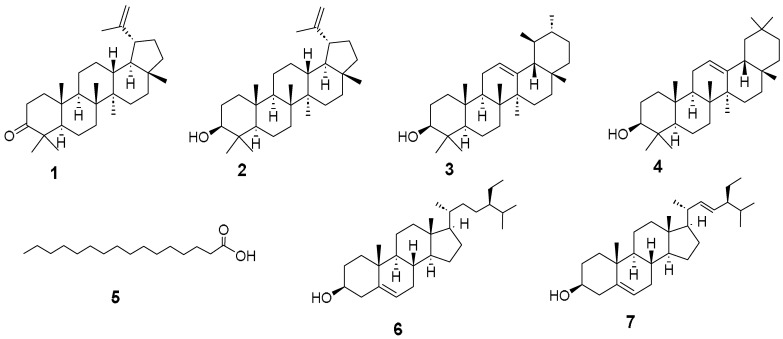
Structures of active compounds isolated from *M. oleifera*.

The hexane fraction was subjected to primary fractionation by open-column chromatography, resulting in 31 (XXXI) primary fractions. As part of a bioassay-guided study, all obtained fractions were tested for bioactivity. Fractions II (with 85% inhibition), FIV (with 81% inhibition), and FVI (with 70% inhibition) were the ones that showed significant inhibition of alpha-glucosidase activity at a concentration of 6.25 µg/mL (concentration previously identified as suitable for initial screening). Subsequent bioassay-guided fractionations of the all-active fractions led to the identification of seven compounds, identified as lupenone (**1**), lupeol (**2**), α-amyrin (**3**), β-amyrin (**4**), palmitic acid (**5**), sitosterol (**6**), and stigmasterol (**7**). The structures of these compounds (Figure 1) were confirmed by spectroscopic analyses, including APCI-HR-MS and NMR techniques (^1^H, ^13^C, DEPT 135, DEPT 90, COSY, NOESY, HMBC, and HMQC). All spectroscopic and spectrometric data obtained were compared with previously reported to confirm the structural assignments [12,13,14,15,16].

#### Spectral Compounds Data

*Lupenone* (**1**): Colorless needles, m.p. 168–170 °C; ^1^H NMR (CDCl_3_, 400 Mhz): δ_H_ 0.68, 0.78, 0.83, 0.91, 0.94, 1.06, 1.72 (each 3H, s, CH3 × 7), 4.56 (1H, s, H-29a), 4.74 (1H, s, H-29b); ^13^C NMR (CDCl_3_, 100 MHz): δ_C_ 212.8 (C-3), 150.4 (C-20), 108.8 (C-29), 59.3 (C-5), 58.0 (C-9), 53.1 (C-18), 42.6 (C-19), 42.2 (C-17), 41.6 (C-4), 41.4 (C-14, 8), 40.4 (C-22), 39.7 (C-1), 36.0 (C-10, 16), 35.6 (C-13), 35.1 (C-2), 33.1 (C-7), 32.3 (C-23), 32.0 (C-24), 30.2 (C-15), 29.7 (C-21), 29.4 (C-12), 22.5 (C-11), 21.0 (C-30), 20.2 (C-28), 18.9 (C-25), 18.5 (C-6), 18.0 (C-26), 15.1 (C-27). APCI-HR-MS *m*/*z* 425.3772 [M + H]^+^ (calculated for C_30_H_49_O, 425.3778).

*Lupeol* (**2**): White powder, m.p. 212–214 °C; ^1^H NMR (CDCl_3_, 400 MHz): δ_H_ 0.72, 0.78, 0.83, 0.91, 0.94, 1.06, 1.70 (each 3H, s, CH_3_ × 7), 3.20 (1H, dd, *J* = 5.4, 10.6 Hz, H-3), 4.56 (1H, s, H-29a), 4.70 (1H, s, H-29b); ^13^C NMR (CDCl_3_, 100 MHz): δ 151.0 (C-20), 109.3 (C-29), 79.0 (C-3), 55.3 (C-5), 50.4 (C-9), 48.3 (C-18), 47.9 (C-19), 43.0 (C-17), 42.8 (C-14), 40.9 (C-8), 40.0 (C-22), 38.1 (C-13), 38.6 (C-4), 38.8 (C-1), 37.2 (C-10), 35.6 (C-16), 34.3 (C-7), 29.9 (C-21), 28.0 (C-23), 27.4 (C-15), 25.1 (C-12), 27.4 (C-2), 20.9 (C-11), 19.3 (C-30), 18.3 (C-6), 18.0 (C-28), 16.1 (C-25), 16.0 (C-26), 15.4 (C-24), 14.6 (C-27). APCI-HR-MS *m*/*z* 427.3931 [M + H]^+^ (calculated for C_30_H_51_O, 427.3934).

*α-amyrin* (**3**) Colorless solid, m.p. 185–187 °C. ^1^H-NMR (CDC_l3_, 400 MHz): δ_H_ 5.16 (t, *J* = 3.6 Hz), 3.23 (dd, *J* = 4.4, 3.9 Hz), 1.96 (td, *J* = 4.4, 13.6 Hz), 1.85 (m), 1.78 (td, *J* d, = 4.9, 13.6 Hz), 1.00 (s), 0.97 (s), 0.94 (s), 0.87 (s), 0.83 (d, *J* = 6.0 Hz), 0.79 (sb) 0.74 (d, *J* = 11.0 Hz). ^13^C-NMR (CDCl_3_, 100 MHz): δ_C_ 38.8 (C-1), 28.6 (C-2), 79.3 (C-3), 38.8 (C-4), 55.2 (C-5), 18.3 (C-6), 32.4 (C-7), 40.6 (C-8), 47.7 (C-9), 36.9 (C-10), 23.3 (C-11), 124.4 (C-12), 139.6 (C-13), 42.1 (C-14), 27.3 (C-15), 26.6 (C-16), 33.7 (C-17), 59.1 (C-18), 39.6 (C-19), 39.7 (C-20), 31.2 (C-21), 41.5 (C-22), 28.1 (C-23), 15.7 (C-24), 15.6 (C-25), 16.8 (C-26), 23.3 (C-27), 28.1 (C-28), 17.5 (C-29), 21.4 (C-30). APCI-HR-MS *m*/*z* 427.3893 [M + H]^+^ (calculated for C_30_H_51_O, 427.3895).

*β-amyrin* (**4**) Colorless solid, m.p. 196–197 °C. ^1^H-NMR (CDCl_3_, 400 MHz): δ_H_ 5.18 (t, *J* = 3.5 Hz), 3.20 (dd, *J* = 4.4, 10.8 Hz), 1.90 (td, *J* = 4.0, 13.6 Hz), 1.81 (m), 1.73 (td, *J* = 4.2, 13.6 Hz), 1.19 (s), 1.09 (s), 0.96 (s), 0.93 (s), 0.92 (d, *J* = 6.4 Hz), 0.84 (s), 0.80 (s), 0.72 (d, *J* = 10.8 Hz). ^13^C-NMR (CDC_l3_, 100 MHz) δ_C_ 38.6 (C-1), 27.2 (C-2), 79.0 (C-3), 38.8 (C-4), 54.9 (C-5), 18.4 (C-6), 32.6 (C-7), 39.8 (C-8), 47.7 (C-9), 36.8 (C-10), 23.5 (C-11), 121.7 (C-12), 145.2 (C-13), 41.7 (C-14), 26.1 (C-15), 27.2 (C-16), 32.5 (C-17), 47.3 (C-18), 46.8 (C-19), 31.2 (C-20), 34.7 (C-21), 37.1 (C-22), 28.1 (C-23), 15.6 (C-24), 15.7 (C-25), 16.9 (C-26), 25.8 (C-27), 28.4 (C-28), 33.7 (C-29), 23.7 (C-30). APCI-HR-MS *m*/*z* 427.3896 [M + H]^+^ (calculated for C_30_H_51_O, 427.3895).

*Palmitic acid* (**5**). White crystalline scales, m.p. 63–64 °C, ^1^H-NMR (CDCl_3_, 400 MHz): δ_H_ 0.84–0.93 (m, CH_3_), 1.25-1.33 (m, 13 × CH_2_), 2.17-2.30 (s, CH_2_, C-2). ^13^C-NMR (CDCl_3_, 100 MHz): δ_C_ 179.0, 33.9 (CH_2_), 32.0 (CH_2_), 29.8-29.1 (CH_2_ × 10), 24.8 (CH_2_), 22.8 (CH_2_), 14.2 (CH_3_). APCI-HR-MS *m*/*z* 257.2486 [M + H]^+^ (calculated for C_16_H_33_O_2_, 257.2481).

*β-Sitosterol* (**6**). Colorless needles; m.p. 134–136 °C, ^1^H-NMR (CDCl_3_): δ_H_ 0.63 (3H, s, CH_3_-18), 0.78 (3H, d, *J* = 6.4 Hz, CH_3_-27), 0.84 (3H, d, *J* = 6.4 Hz, CH_3_-26), 0.88 (3H, t, *J* = 7.2 Hz, CH_3_-29), 0.94 (3H, d, *J* = 6.4 Hz, CH_3_-21), 1.01 (3H, s, CH_3_-19), 3.52 (1H, m, H-3), 5.34 (1H, dd, *J* = 1.7, 3.5 Hz, H-6); ^13^C-NMR (CDCl_3_): δ_C_ 37.2 (C-1), 31.8 (C-2), 71.9 (C-3), 42.5 (C-4), 140.9 (C-5), 121.8 (C-6), 32.1 (C-7), 31.8 (C-8), 50.1 (C-9), 36.7 (C-10), 21.2 (C-11), 39.7 (C-12), 42.4 (C-13), 56.7 (C-14), 24.4 (C-15), 28.3 (C-16), 56.2 (C-17), 11.8 (C-18), 19.5 (C-19), 36.3 (C-20), 18.9 (C-21), 34.0 (C-22), 26.4 (C-23), 45.8 (C-24), 29.4 (C-25), 19.8 (C-26), 19.4 (C-27), 23.2 (C-28), 12.3 (C-29). APCI-HR-MS *m*/*z* 415.3939 [M + H]^+^ (calculated for C_29_H_51_O, 415.3934).

*Stigmasterol* (**7**). Colorless crystalline solid, m.p. 171–173 °C. ^1^H-NMR (CDCl_3_, 400 MHz): δ_H_ 5.33 (m, H-6), 5.15 (dd, *J* = 15.2, 8.0 Hz, H-22), 5.02 (dd, *J* = 15.2, 8.0 Hz, H-23), 3.28 (m, H-3), 0.90 (d, *J* = 6.4 Hz, CH_3_-21), 0.83 (d, *J* = 6.5 Hz, CH_3_-26), 0.84 (t, *J* = 7.0 Hz, CH_3_-29), 0.81 (d, *J* = 6.4 Hz, CH_3_-27), 0.80 (s, CH_3_-19), 0.65 (s, CH_3_-18). ^13^C-NMR (CDCl_3_, 100 MHz): δ_C_ 140.9 (C-5), 138.4 (C-22), 129.4 (C-23), 121.7 (C-6), 71.9 (C-3), 57.0 (C-14), 56.0 (C-17), 51.3 (C-24), 50.3 (C-9), 42.5 (C-13), 42.2 (C-4), 40.5 (C-20), 39.7 (C-12), 37.5 (C-1), 36.6 (C-10), 32.2 (C-8), 32.0 (C-25), 31.9 (C-7), 31.8 (C-2), 28.9 (C-16), 25.4 (C-28), 24.4 (C-15), 21.2 (C-27), 21.1 (C21), 21.0 (C-11), 19.4 (C-19), 19.0 (C-26), 12.4 (C-18), 12.0 (C-29). APCI-HR-MS *m*/*z* 413.3787 [M + H]^+^ (calculated for C_29_H_49_O, 413.3783).

### 2.2. Characterization of Alpha-Glucosidase Enzyme Inhibition by Isolated Compounds

Compounds **1**–**7** exhibited concentration-dependent inhibition of the alpha-glucosidase enzyme, with IC_50_ values of 0.72, 1.05, 2.13, 1.22, 240.20, 18.70, and 163.10 µM, respectively. Notably, all compounds displayed greater potency against alpha-glucosidase than acarbose (the positive control), having an IC_50_ value of 241.6 µM. Upon examining the active compounds’ chemical structures, we noticed a remarkable similarity in their structures, as six active compounds shared a biogenetic origin and had a common initial base structure, derived from a cyclopentanoperhydrophenanthrene nucleus. This suggests that the structure of this core might be responsible for enzyme inhibition. However, it is important to understand that other factors can affect the strength of enzyme inhibition. These factors include the type and position of functional groups linked to the central core, the molecule’s stereochemistry, the overall polarity or hydrophobicity of the compound, the ability of their functional groups to form hydrogen bonds, and the presence of electron-donating or electron-withdrawing groups that can influence molecular interactions. Additionally, the three-dimensional molecule arrangement and how well it fits into the enzyme’s active site can also play an important role.

### 2.3. Mode of Inhibition of α-Glucosidase for Compounds ***1***, ***6***, and ***7***

**Figure 2:**
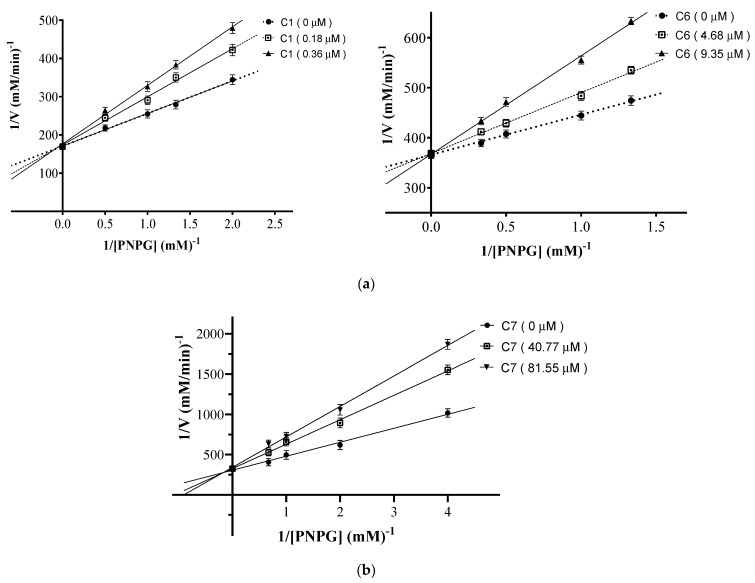
Lineweaver-Burk plots of alpha-glucosidase inhibition of (**a**) compounds **1**, **6**, and (**b**) compound **7** at different substrate concentrations.

We previously determined that compounds **2**–**5** act as competitive inhibitors of the enzyme alpha-glucosidase [15,16,17]. To have complete information on the mode of inhibition of all active compounds isolated from *M. oleifera,* we analyzed compounds **1**, **6**, and **7**. In Figure 2, the Lineweaver–Burk plots are shown, presenting the inhibitory activities of compounds **1**, **6**, and **7** on alpha-glucosidase using two concentrations of inhibitors and in the absence of them and using various substrate concentrations (PNPG). The results in Figure 2 indicates that all compounds follow similar reversible competitive inhibition patterns, with lines intersecting at the same y-intercept as the enzyme without any inhibitors. This suggests that compounds **1**–**7** bind to the alpha-glucosidase active site or the complex of the enzyme with its normal substrate. It is worth mentioning that acarbose, the positive control, also showed competitive inhibition.

### 2.4. Docking Study for Compounds ***1*** and ***2***

Molecular docking studies are important theoretical analyses used to predict how a specific ligand binds to a protein of a known three-dimensional structure [18]. In our previous research, we discovered that the presence of a hydroxyl group at the 3-β position of the pentacyclic triterpene core is crucial for the inhibition of alpha-glucosidase enzyme because the hydrogen of this functional group can form hydrogen bridge bonds by its positive partial charge, thus attracting the electron density of a nearby electro-negative atom of amino acids present in the alpha-glucosidase structure [16,17]. However, in compound **1** isolated in this work, the hydroxyl group was in a more oxidized state, so it was interesting to explore the effect of this change on the biological activity detected. For this purpose, we decided to perform a molecular modeling analysis of compounds **1** and **2** to find evidence that would help us understand whether the difference in IC_50_ of each compound was linked to the functional group present at position 3 of the pentacyclic nucleus of the triterpene.

**Figure 3:**
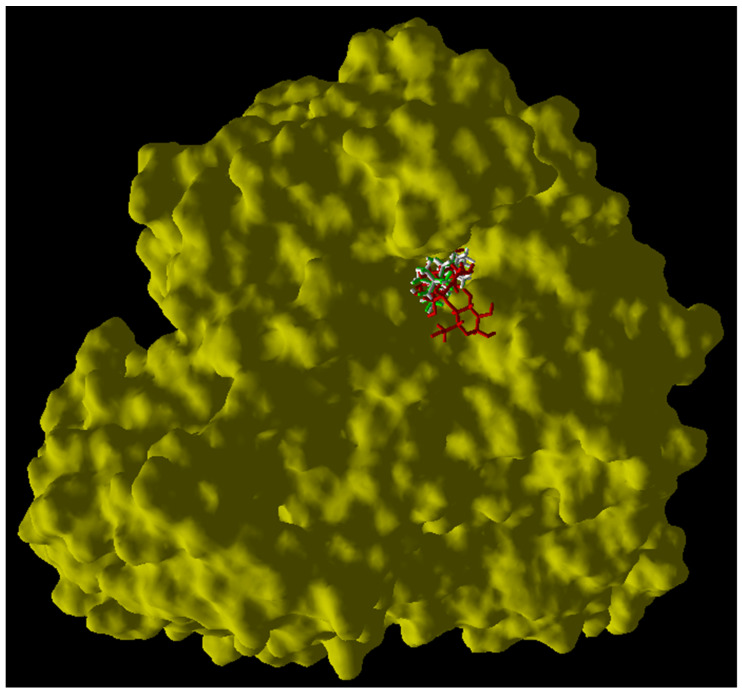
Superposition of docking poses of compound **1** (in green), compound **2** (in white), and acarbose (in red).

**Figure 4:**
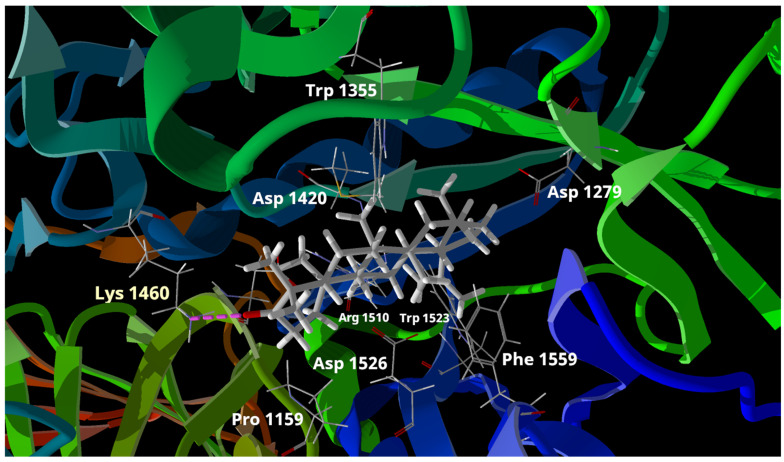
Docking poses of compound **1**. Interaction with Lys 1460 is highlighted. The image also shows the structures of the amino acid residues of the protein located at a distance of less than 2 Å and interacting electrostatically with compound **1**.

**Figure 5:**
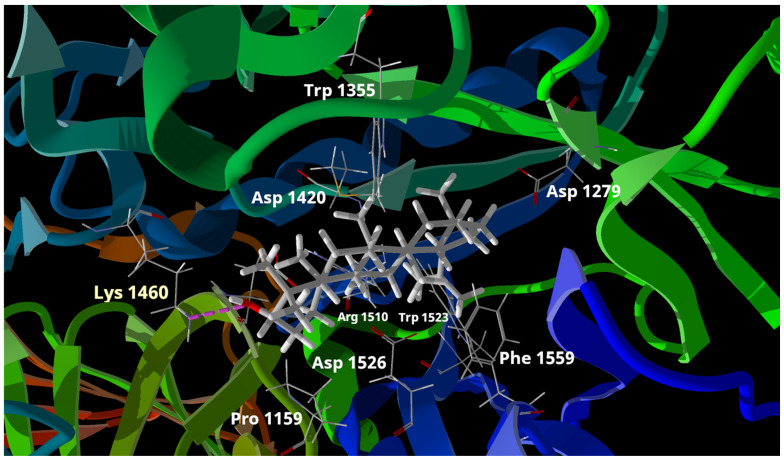
Docking poses of compound **2**. Interaction with Lys 1460 is highlighted. The image also shows the structures of the amino acid residues of the protein located at a distance of less than 2 Å and interacting electrostatically with compound **2**.

For the molecular docking study, we used the enzyme human intestinal alpha-glucosidase deposited in GenBank with the code PDB: 3TOP. After the analysis, the results indicate that both compounds primarily engage in hydrophobic interactions upon binding to the enzyme. Figure 3 illustrates the overlapping docking poses of the compounds and acarbose within the binding site. Compounds **1** and **2** primarily interacted with specific amino acids in the active site of the alpha-glucosidase enzyme through hydrophobic interactions. It is noteworthy that despite compound **1** having a carbonyl group at position 3 and compound **2** having a hydroxyl group at the same position, both compounds significantly interacted with Lys 1460 through hydrogen bonds (Figure 4 and Figure 5). This specific interaction seems to play a critical role in the enzymatic inhibition demonstrated by both compounds. The most favorable calculated pose (Figure 4 and Figure 5) for each compound involved a hydrogen bonding interaction between the 3β-OH (compound **2**) and Lys 1460 (enzyme), as well as the 3-carbonyl (compound **1**) and Lys 1460 (enzyme). These poses consistently yielded lower scores than the other poses of the same compounds where only ionic interactions were observed. Furthermore, a correlation was observed between the IC_50_ values and Rerank scores, where lower IC_50_ values indicated better inhibitory activity, and lower Rerank scores indicated stronger interaction with the enzyme. Table 1 summarizes the IC_50_ values (alpha-glucosidase) and Rerank scores (molecular docking). Considering the comprehensive analysis of all the evidence, it can be inferred that oxygen in the 3 position and its oxidation state accounted for the higher potency of compound **1** than compound **2**.

In a previous study published in 2021, Zhao et al. [19] reported a molecular docking analysis of the interaction between lupenone and the enzyme alpha-glucosidase. However, some of the binding interactions observed in that previous study by Zhao et coworkers differed from those found in the current work, highlighting that lupenone did not form hydrogen bonds with any of the amino acids of α-glucosidase. Yet, both works showed that hydrophobic interactions play a major role in enzymatic inhibition. In this sense, it is important to note that the 2021 study was performed with AutoDock software (version 4.2), while the current study employed the Molegro Virtual Docker (MVD version 6.0.1) program. It is well established that AutoDock and MVD, differ in their underlying algorithms, scoring functions, user interfaces, and specific capabilities [20,21,22]. For this reason, some studies have emphasized the importance of using multiple molecular modeling software tools to obtain complementary results and to gain a more comprehensive understanding of the molecular-level interactions between ligands and proteins [20,21,22]. It is a fact that the use of software with different docking algorithms and scoring functions can help us to evaluate and infer better possible binding modes and affinities, ultimately leading to a more robust and reliable interpretation of molecular interactions.

Consequently, molecular docking research is a theoretical study that approximates the interaction between ligands and receptors, and the results may vary depending on the algorithm used. Therefore, this study complements existing published studies.

### 2.5. GC-MS-Based Metabolomic Analysis

To our knowledge, there have been no reports on the secondary metabolites produced by the mangrove species *M. oleifera*. To obtain comprehensive information about the chemical composition of *M. oleifera*, we conducted a metabolomic analysis of each fraction using gas chromatography coupled with mass spectrometry. The initial metabolomic analysis comprised the establishment of the optimal chromatographic conditions for better peak separation corresponding to the compounds present in each fraction analyzed. For this purpose, we evaluated several temperature gradient programs to identify the optimal one for each sample. This optimization step was crucial to ensure a more efficient separation and detection of the metabolites contained in the analyzed fractions, as well as to obtain a more complete chemical profile, facilitating the identification and characterization of the metabolites by allowing better matches with the database. To validate the method for compound identification, three previously isolated and identified compounds (lupenone, lupeol, and sitosterol) were used as standards and injected into the system. By establishing a correlation between the mass identification and retention time between standard compounds and the same compounds detected in the chromatogram of the fractions, we were able to validate the identification of the volatile components present in the fractions of *M. oleifera*.

**Figure 6:**
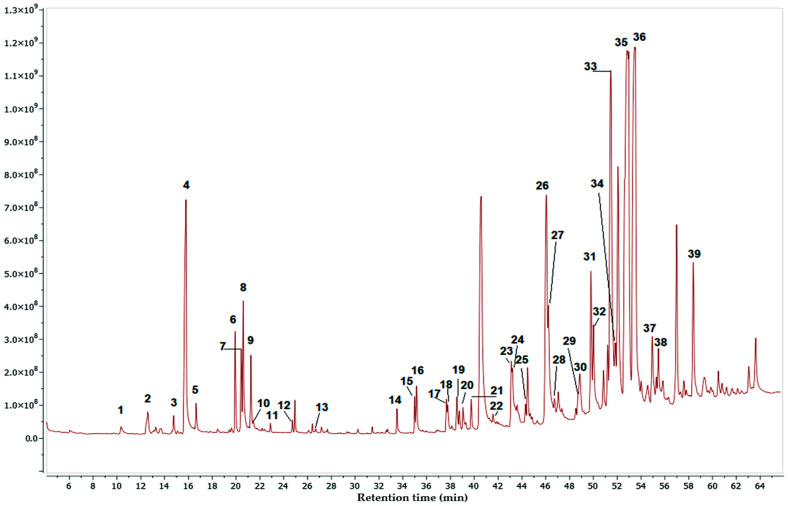
GC-MS chromatogram for the hexane fraction of *M. oleifera*.

**Figure 7:**
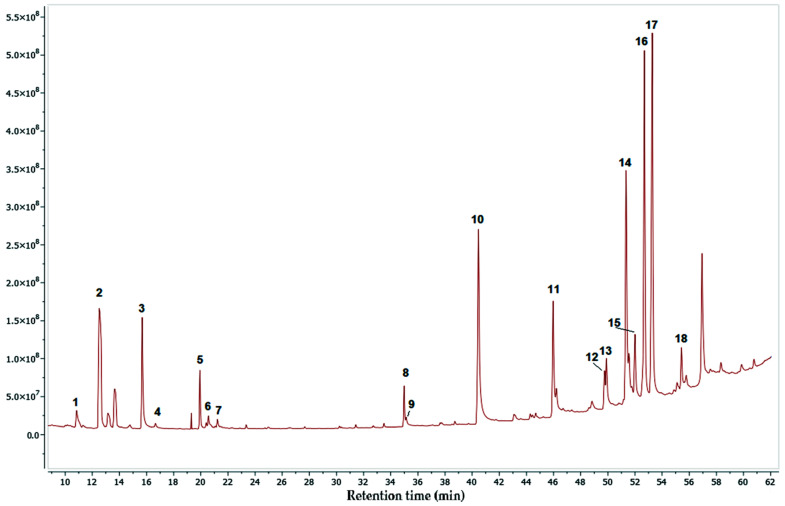
GC-MS chromatogram for the DCM fraction of *M. oleifera*.

**Figure 8:**
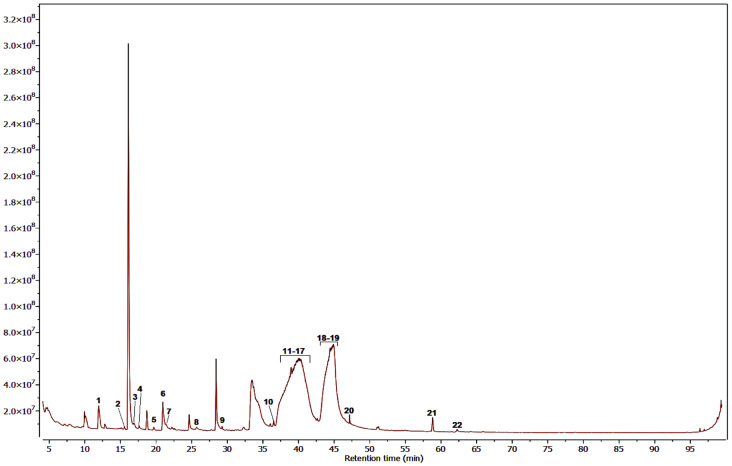
GC-MS chromatogram for the MeOH fraction of *M. oleifera*.

Figure 6, Figure 7 and Figure 8 show the chromatograms obtained under the best conditions tested. The hexane and dichloromethane fractions were successfully analyzed using the same temperature gradient program. However, the methanol fractions presented more analytical challenges, primarily due to the high polarity of their constituents and the presence of a group of highly similar compounds, which were subsequently identified as compounds of the sugars type. Despite the analytical obstacles posed by the methanol fraction, we obtained a significant list of identified compounds, which allowed us to increase the overall picture of the diverse range of metabolites produced by this mangrove plant species.

Table 2, Table 3 and Table 4 display lists of compounds detected with an identification percentage higher than 90 percent. In the case of the hexane fraction, 39 compounds were identified, with the presence of palmitic acid, stigmasterol, sitosterol, taraxerol, lupenone, and lupeol. These compounds have been proven to be alpha-glucosidase inhibitors. On the other hand, only 18 compounds could be detected with high certainty in the dichloromethane fraction. It was found that most of the compounds detected in the hexane fraction were also present in this fraction. Finally, the Methanol fraction contained approximately 22 compounds, including several simple phenolic compounds common plant metabolites, including catechol, hydroquinone, resorcinol, and pyrogallol. In addition, this fraction also contained a complex mixture of compounds that were challenging to separate effectively. As a result, it was necessary to employ different strategies to identify the individual components within this complex mixture. This difficulty arose because the chromatographic peaks corresponding to these compounds were quite broad, which led to lower matching percentages during the compound identification process, as shown in Table 4. It is important to note that although the methanol fraction could be analyzed using analytical methods more affine with its polarity since our main objective was to obtain the active compounds from this mangrove plant, we did not perform an in-depth investigation on the composition of this fraction.

Upon analyzing the identified compounds in the active fractions, we observed that the hexane fraction had a more complex composition of volatile compounds, with 39 compounds detected, while the dichloromethane fraction only had 18 compounds. This result was expected since the hexane fraction, being less polar, was anticipated to contain a greater diversity of these compounds. Surprisingly, both fractions shared a significant percentage of compounds, indicating that the extraction process may not have been sufficiently prolonged to extract all soluble substances in each solvent. However, when we examined the inhibitory concentration 50 (IC_50_), it became evident that the hexane fraction contained a higher concentration of active compounds.

### 2.6. Molecular Networking

**Figure 9:**
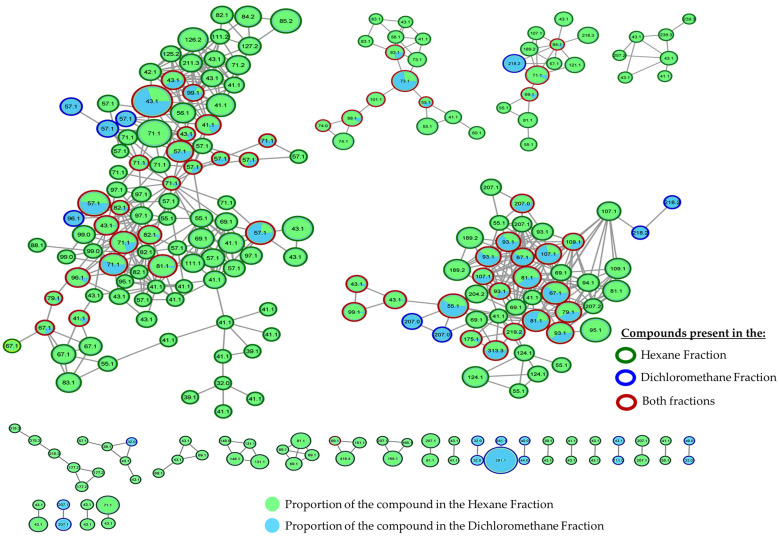
Molecular networks filtered by the relative abundance of ions in the hexane and dichloromethane fractions. The node size represents the relative abundance of ions. The composition of compounds in the hexane fraction is indicated in green, while the composition in the dichloromethane fraction is depicted in blue.

**Figure 10:**
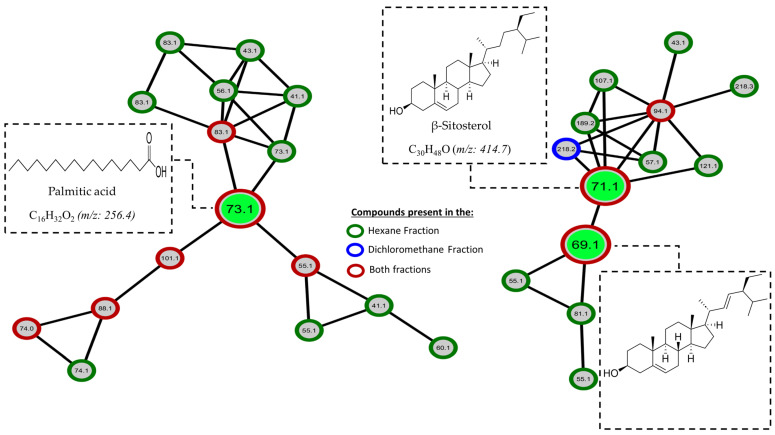
Molecular networks filtered by the relative abundance of ions in the hexane and dichloromethane fractions. Expansion of the cluster where active compounds **5**–**7** are present in green.

**Figure 11:**
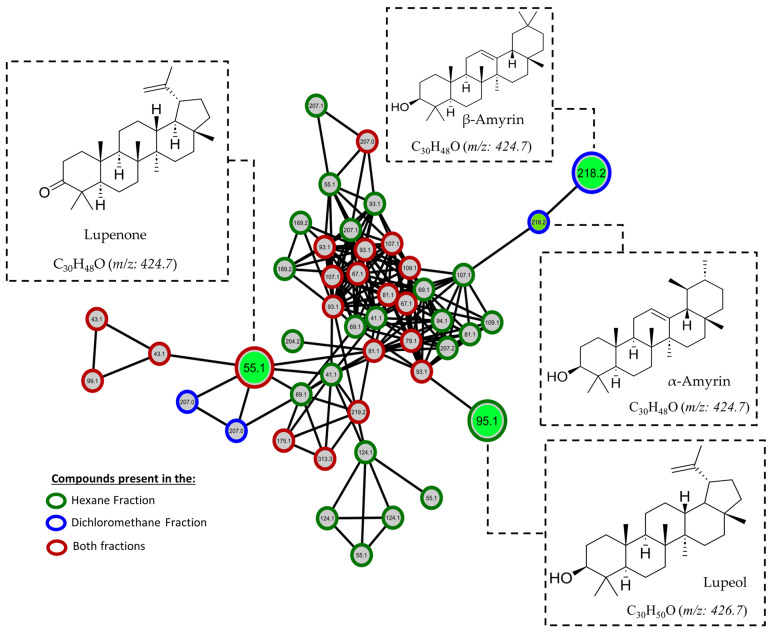
Molecular networks filtered by the relative abundance of ions in the hexane and dichloromethane fractions. Expansion of the cluster where active compounds **1**–**4** are present in green.

Nowadays, many tools allow us to make a detailed metabolomics analysis using the data obtained in a mass spectrometer, such as the molecular networks generated with GNPS (Global Natural Products Social Molecular Networking), which gives us valuable information about the chemical diversity and relationships between molecules in a given data set [23]. In other words, this tool helps us to analyze molecular similarities and differences, identify related compounds, and explore possible structural connections. To take advantage of this tool and have a different view of our data, we proceeded to perform a detailed analysis in GNPS. The GNPS platform generated the mass spectrometry molecular network with the data initially obtained by GC-EI/MS. Figure 9 shows the molecular network generated with the compounds produced by *M. oleifera* from hexane and dichloromethane fractions; it can be observed that these compounds are grouped according to their similar chemical characteristics. Each cluster represents a specific group of secondary metabolites produced by this plant, highlighting the presence of triterpenes, sterols, and fatty acids. It is important to note that numerous unique or unidentified compounds do not match known structures or are absent from the database. These compounds may represent new or uncommon natural products. This discovery highlights the significance of further research focused on studying and isolating these compounds, as it could potentially lead to the isolation of new bioactive substances in *M. oleifera*. Figure 10 and Figure 11 provide a closer look at the clusters where the active compounds isolated in this study are located. These compounds are the most abundant and relatively easier to isolate due to their lower chemical complexity. Although many other compounds within the same cluster could also possess activity, verifying this was challenging due to their limited quantities.

**Figure 12:**
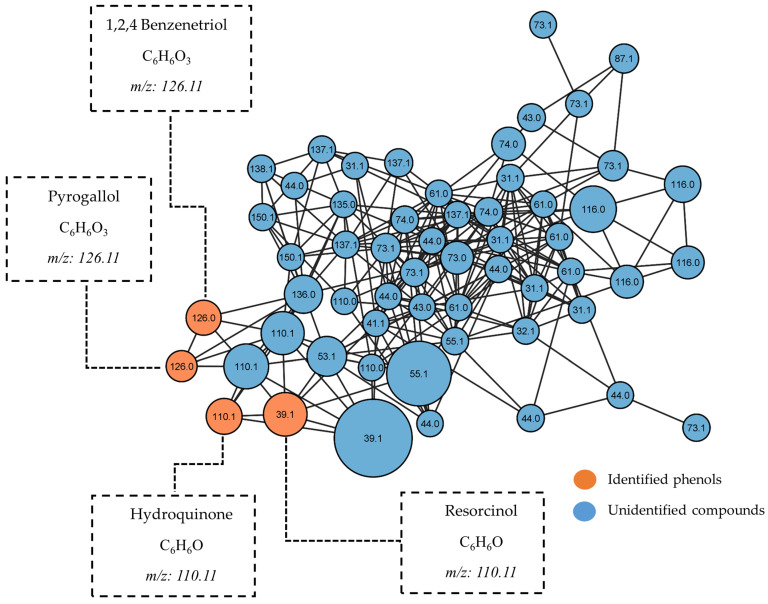
Molecular networks filtered by the relative abundance of ions in the methanol fraction. The node size represents the relative abundance of ions.

Figure 12 shows the molecular network generated with the compounds contained in the methanol fraction. In this particular fraction, the data analysis revealed the presence of a single dominant cluster, which was primarily composed of a series of simple phenolic compounds. The key constituents identified within this cluster included hydroquinone, resorcinol, syringol, and 1,2,4-benzenetriol. On the other hand, several nodes within the dataset did not match any known compounds in the public database integrated with the GNPS platform. These unidentified nodes could represent either unreported known compounds in the GNPS database or potentially novel compounds that have not yet been reported. Further investigation of these unmatched nodes, potentially through the use of advanced spectroscopic techniques and comparison with reference standards, could lead to the discovery of novel natural products derived from this mangrove species.

## 3. Experimental

### 3.1. Plant Material and Extract Preparation

*M. oleifera* (Fabaceae) leaves were collected at Pedregal Port, David, Chiriquí. Alejandro de Sedas identified this plant. A voucher specimen (110761) has been deposited at the University of Panama Herbarium. Dried leaves (890 g) were sequentially extracted by maceration process using a series of solvents in increasing order of polarity (Hexanes (Hex), Dichloromethane (DCM), Methanol (MeOH)). Fractions were concentrated to a semisolid paste using a Rotatory Evaporator (R-215, Buchi, Switzerland) to obtain 13,375 g of hexanes dried fraction, 16.11 g of dichloromethane fraction, and 23.92 g of methanol fraction.

### 3.2. Isolation of Compounds

Initially, the Hex fraction was treated with activated charcoal to remove excess of chlorophyll and apolar pigments from the extract and facilitate the fractionation process. The effectiveness of the process was evident in transforming the initial fraction’s dark green color into a brownish-yellow hue color. The Hex fraction was fractionated by column chromatography on silica gel (100 g). The column was eluted with Hex, followed by a gradient of Hex:DCM (1:0 → 0:1), and finally with a gradient of DCM:MeOH (1:0 → 1:1). Altogether, 116 fractions (100 mL each) were collected and combined according to their TLC profiles to yield 21 primary fractions (FI to FXXI).

Fraction FII (94.1 mg) eluted with Hex:DCM (8:2), was purified by normal phase HPLC (Sphereclone silica 250 × 10 mm column, isocratic elution of 90% hexanes:10% DCM, UV detector at 254 nm, flow of 1 mL/min) to afford 71 mg of lupenone (**1**). Fraction FIII (101.4 mg) eluted with Hex:DCM (7:3), was purified by normal phase HPLC (Sphereclone silica 250 × 10 mm column, isocratic elution of 80% hexanes:20% DCM, UV detector at 254 nm, flow of 1 mL/min) to afford 49 mg of lupeol (**2**). Fraction FIV (1001.4 mg) eluted with Hex:DCM (6:4), was purified by normal-phase HPLC (Sphereclone silica 250 × 10 mm column, isocratic elution of 70% hexanes:30% DCM, UV detector at 254 nm, flow of 1 mL/min) to afford 51 mg of β-sitosterol (**3**), 13 mg of stigmasterol (**4**), 8 mg of palmitic acid (**5**). Finally, Fraction FVI (101.4 mg) eluted with Hex:DCM (4:6), was purified by normal phase HPLC (Sphereclone silica 250 × 10 mm column, isocratic elution of 50% hexanes:50% DCM, UV detector at 254 nm, flow of 1 mL/min) to afford 2 mg of α-amyrine (**7**), and 4 mg of β-amyrine (**7**). The purification of the compounds was carried out on Agilent (Santa Clara, CA, USA) 1100 HPLC system equipped with a quaternary pump, a diode array detector.

### 3.3. Inhibition of Alpha-Glucosidase Assay

The assay was performed in 96-well plates. Both test samples and the positive control were dissolved in DMSO or MeOH. Test samples (20 μL) were added to their respective wells containing 150 μL of the enzyme (from *Saccharomyces cerevisiae* purchased from Sigma-Aldrich Co., St. Louis, MO, USA) solution (32 mU/mL), followed by incubation at 37 °C for 7 min. Subsequently, 150 μL of PNPG (4-Nitrophenyl-β-D-glucopyranoside, 2 mM) was introduced into each well, and the plate was further incubated at 37 °C for 20 min. The resulting absorbance, corresponding to the *p*-nitrophenol released during the reaction, was measured at 400 nm. All assays were performed in duplicate [16,24]. The activity of the samples was calculated as a percentage relative to the control (DMSO or MeOH, instead of the sample solution), using the following equation:

%Inhibition=(∆ Acontrol−∆ Asample)∆ Acontrol×100%

Δ*A_control_*: absorbance of the control—absorbance of the blank

Δ*A_sample_*: absorbance of the sample—absorbance of the sample blank.

The concentration necessary to achieve a 50% inhibition of enzyme activity (IC_50_) was calculated through regression analysis. Data obtained from alpha-glucosidase inhibition assays were expressed as the mean ± standard deviation (SD) of three replicates. Statistical analysis was performed using Excel 2019 (Microsoft, Seattle, WA, USA). Finally, a one-way analysis of variance (ANOVA) followed by Tukey’s post-test was used to evaluate possible differences between the means of each data. To determine significant differences, statistical significance was defined as *p*-values ≤ 0.05 [25].

### 3.4. Kinetics of Alpha-Glucosidase Inhibition

To determine the mode of inhibition of the compounds, fixed amounts of alpha-glucosidase were incubated with increasing concentrations of PNPG at 37 °C for 15 min, both in the absence and presence of inhibitors. Subsequently, the reactions were stopped, the sample absorbances were measured, and then the values obtained were analyzed using the Lineweaver–Burk plot. For this assay, three evaluations were performed, one in the absence of the inhibitor and the other using different concentrations of the inhibitors (at concentrations equivalent to their respective IC_50_ values). Again, the evaluations of each compound were performed in triplicate [16,17].

### 3.5. Docking Study

The ligands were constructed using Spartan’10, and their geometry was optimized using the MMFF force field [26]. A protein–ligand docking study was performed using the crystal structures of the C-terminal domain of human intestinal α-glucosidase (PDB:3TOP) [27], obtained from the Protein Data Bank [28]. Molecular docking calculations were performed using Molegro Virtual Docker v. 6.0.1 [29], showing excellent results in previous evaluations. Before the docking analysis, all solvent molecules and the co-crystallized ligand included in the structure obtained from the Protein Data Bank were removed. Considering the results of enzyme kinetics, for the search a sphere with a radius of 10 Å was placed at the active site as a binding site [15,16,17].

Protonation states and charge assignments for the protein were determined using standard templates from the Molegro Virtual Docker program, requiring no additional charge settings. Various orientations of the ligands were explored and ranked based on their energy scores. The RMSD threshold for multiple clustered poses was set to <1.00 Å. The docking parameters were configured with a maximum of 5000 iterations, a simplex evolution population size of 100, and a minimum of 50 runs for each ligand. To assess the effectiveness of the docking procedure in finding low-energy solutions, the co-crystallized ligand (acarbose) was included in the analysis. The top-ranking score was obtained, and the RMSD between the pose and the corresponding crystal coordinates was calculated. As the RMSD result obtained was less than 2 Å, it could be concluded that the molecular docking simulation methodology was adequate. The MolDock and ReRank scores presented the energy needed in the receptor–ligand bond [30]. The lowest energy visualized the best binding pose between the protein’s ligand and amino acid residue.

### 3.6. GC-MS-Based Metabolomic Analysis

The untargeted metabolomic analysis of all fractions from *M. oleifera* was conducted using an Agilent 8890 Gas Chromatograph coupled with an Agilent 5977C mass spectrometer. Separation was performed using a HP-5MS capillary column (30 m length, 25 mm ID, 0.25 μm df, Agilent, Santa Clara, CA, USA), with high-purity helium as the carrier gas flowing at a constant rate of 1.1 mL/min. For hexane and dichloromethane fractions, the GC temperature program initiated at 150 °C, followed by oven temperature ramps of 3 °C/min to 250 °C and 2 °C/min to 305 °C. A final 5 min hold was maintained at 305 °C. For methanol fraction, the GC temperature program initiated at 80 °C, followed by oven temperature ramps of 2 °C/min to 140 °C, 1 °C/min to 160 °C, 1 °C/2 min to 180 °C, and 20 °C/min to 305 °C. A final 5 min hold was maintained at 305 °C. The electron impact (EI) ion source was held at 250 °C with a filament bias of −70 V. Full scan mode (*m*/*z* 30–600) was employed, and data acquisition was performed at a rate of 20 spectra/second in the MS setting.

### 3.7. Molecular Networking

The GC-EI/MS data were initially processed using the MassHunter software (version 10.1.49) from Agilent. Mass spectrometry molecular networks were generated using the GNPS platform (http://gnps.ucsd.edu, accessed on 12 May 2024) [23,31]. Since the mass data obtained from the EI experiments did not have pre-selected precursor ions (referred to as the DIA acquisition format), spectral deconvolution was necessary. For this purpose, the GC-MS data were analyzed and processed using the MzMine 2.53 package [31].

The raw data were submitted for processing to the spectral network algorithm (GNPS), and they were fitted using the following parameters: a fragment ion mass tolerance of 0.5 Da, a minimum of 5 matched peaks, and a score threshold of 0.7. In terms of search options, the following parameters were used: a gold library class, the top history per spectrum was selected as 10, and both the NIST20 and GNPS spectral libraries were utilized. The minimum pair cosine similarity was set to 0.85 and the network topK to 15 in the advanced network options. For more detailed information about the network on GNPS, visit: https://gnps.ucsd.edu/ProteoSAFe/status.jsp?task=32fd298e911f411b96d8e7460070aff4 for hexane and dicloromethane fractions (accessed on 8 May 2024) and https://gnps.ucsd.edu/ProteoSAFe/status.jsp?task=f0d9a5ea5ce74b519992a21ed06fe4e7 for methanol fraction (accessed on 3 June 2024). The network visualization was performed using Cytoscape v.3.4.3. Node colors and sizes were assigned based on the metadata files, and the thickness of the edges represented the cosine similarity scores, with thicker lines indicating a higher degree of similarity [31,32,33].

## 4. Conclusions

In conclusion, we isolated seven active compounds from the endemic mangrove plant *M. oleifera*. Although these compounds have been reported in other plants, this is the first report of their production by *M. oleifera*. Among them, we identified four pentacyclic triterpenes that displayed potent inhibitory activity against alpha-glucosidase, an enzyme associated with diabetes. These triterpenes exhibited a competitive type of inhibition alpha-glucosidase and demonstrated higher inhibitory activity than acarbose. This suggests that *M. oleifera* could be a promising alternative for managing blood sugar levels in individuals with diabetes. Additionally, our metabolomic analysis identified 64 compounds within the *M. oleifera* fractions, providing novel insights into the plant’s composition. Furthermore, utilizing the GNPS tool for detailed data analysis, we successfully grouped compounds in the analyzed fractions based on similarities and fragmentation patterns, revealing families of compounds. Notably, specific compound families displayed similar MS profiles, with the active compounds positioned within the same node, indicating their chemical relatedness.

## Figures and Tables

**Table 1:** MolDock and Rerank scores obtained during docking analysis.

Compound	IC_50_ (μM)	MolDock Score	Rerank Score	Interaction with Lys 1420
**1**	0.72	−111.929	44.4241	Hydrogen bond
**2**	1.05	−111.304	50.206	Hydrogen bond

**Table 2:** Chemical composition of the hexane fraction of *M. oleifera*.

No.	RT (min)	Compound	P%	MW	MF	L
1	10.36	Tetradecanoic acid	98	228.37	C_14_H_28_O_2_	Nist20
2	12.58	Neophytadiene	96	278.5	C_20_H_38_	Nist20
3	14.77	Methyl Palmitate	98	270.5	C_17_H_34_O_2_	Nist20
4	15.79	Palmitic acid	99	256.42	C_16_H_32_O_2_	Nist20
5	16.66	Ethyl palmitate	98	284.5	C_18_H_36_O_2_	Nist20
6	19.92	Phytol	91	296.5	C_20_H_40_O	Nist20
7	20.44	Isolinoleic acid	91	280.4	C_18_H_32_O_2_	Nist20
8	20.60	6-octadecanoic acid	94	282.5	C_18_H_34_O_2_	Nist20
9	21.24	Stearic acid	95	284.5	C_18_H_36_O_2_	Nist20
10	21.46	1-hexacosanol	90	382.7	C_26_H_54_O	Nist20
11	22.89	1-docosane	92	310.61	C_22_H_46_	Nist20
12	24.74	Dotriacontane	99	450.9	C_32_H_66_	Nist20
13	26.68	Hexyl palmitate	96	340.6	C_22_H_44_O_2_	Nist20
14	33.51	1,21 docosadiene	99	306.6	C_22_H_42_	Nist20
15	34.99	Octacosyl acetate	99	452.8	C_30_H_60_O_2_	Nist20
16	35.16	Heneicosane	99	296.6	C_21_H_44_	Nist20
17	37.67	9-hexacosene	99	364.7	C_26_H_52_	Nist20
18	37.78	Tetracosane	96	336.6	C_24_H_48_	Nist20
19	38.54	Squalene	95	410.7	C_30_H_50_	Nist20
20	39.07	Alpha-tocospiro B	91	448.7	C_28_H_48_O_4_	Nist20
21	39.75	Alpha-tocospiro A	93	462.7	C_29_H_50_O_4_	Nist20
22	41.58	Delta-tocopherol	96	402.65	C_27_H_46_O_2_	Nist20
23	43.10	1-nonadecene	99	266.5	C_19_H_38_	Nist20
24	43.20	Triacontane	99	422.8	C_30_H_62_	Nist20
25	44.31	Pentadecanal	91	226.40	C_15_H_30_O	Nist20
26	46.05	Beta-amyrene (olean-12-ene)	90	410.7	C_30_H_50_	Nist20
27	46.22	D-Friedoolean-14-ene	90	410.7	C_30_H_50_	Nist20
28	46.75	Dl-alpha-tocopherol	92	430.7	C_29_H_50_O_4_	Nist20
29	48.72	Z-14-Nonacosene	97	406.8	C_28_H_58_	Nist20
30	48.86	Campesterol	99	400.7	C_29_H_48_O	Nist20
31	49.79	Stigmasterol	99	412.7	C_29_H_48_O	Nist20
32	50.01	Triacontanal	90	436.8	C_30_H_60_O	Nist20
33	51.46	Sitosterol	96	414.7	C_29_H_50_O	Nist20
34	51.83	alpha-amyrine	90	426.7	C_30_H_50_O	Nist20
35	52.97	Lupenone	99	424.7	C_30_H_48_O	Nist20
36	53.46	Lupeol	91	426.7	C_30_H_50_O	Nist20
37	54.94	Sitostenone	91	412.7	C_29_H_48_O	Nist20
38	55.45	Dotriacontanal	95	464.8	C_32_H_64_O	Nist20
39	58.38	Phytyl stearate	92	563.0	C_38_H_74_O_2_	Nist20

MW: Molecular Weight, P%: Probability Percentage, MF: Molecular Formula, L: Library.

**Table 3:** Chemical composition of the DCM fraction of *M. oleifera*.

No.	RT (min)	Compound	P%	MW	MF	L
1	10.84	Loliolide	99	196.2	C_11_H_16_O_3_	Nist20
2	12.52	Neophytadiene	89	278.5	C_20_H_40_O	Nist20
3	15.68	Palmitic acid	99	256.4	C_16_H_32_ O_2_	Nist20
4	16.66	Ethyl palmitate	95	284.5	C_18_H_36_O_2_	Nist20
5	19.34	Phytol	97	296.5	C_20_H_40_O	Nist20
6	20.56	6-Octadecenoic acid	95	282.5	C_18_H_34_O_2_	Nist20
7	21.23	Octadecanoic acid	99	284.48	C_18_H_36_O_2_	Nist20
8	34.99	Octacosyl acetate	99	452.8	C_30_H_60_O_2_	Nist20
9	35.14	Tricosane	95	324.6	C_23_H_48_	Nist20
10	40.47	1-tetracosene	99	334.6	C_24_H_48_	Nist20
11	45.97	Eicosane	92	282.5	C_20_H_42_	Nist20
12	49.76	Stigmasterol	91	412.7	C_29_H_48_O	Nist20
13	49.90	Triacontanal	97	436.8	C_30_H_60_O	Nist20
14	51.34	Sitosterol	99	414.7	C_29_H_50_O	Nist20
15	52.00	Beta-amyrin	97	426.7	C_30_H_50_O	Nist20
16	52.69	Lupenone	99	424.7	C_30_H_48_O	Nist20
17	53.28	Lupeol	96	426.7	C_30_H_50_O	Nist20
18	55.43	Dotriacontanal	93	464.8	C_32_H_64_O	Nist20

MW: Molecular Weight, P%: Probability Percentage, MF: Molecular Formula, L: Library.

**Table 4:** Chemical composition of the MeOH fraction of *M. oleifera*.

No.	RT (min)	Compound	P%	MW	MF	L
1	11.93	Catechol	96	110.11	C_6_H_6_O_2_	Nist20
2	15.57	Hydroquinone	90	110.11	C_6_H_6_O_2_	Nist20
3	16.88	Resorcinol	93	110.11	C_6_H_6_O_2_	Nist20
4	17.62	2-methoxy-4-vinylphenol	86	150.17	C_9_H_10_O_2_	Nist20
5	19.68	Syringol	89	154.16	C_6_H_6_O_3_	Nist20
6	20.94	1,2,4-Benzenetriol	98	126.11	C_6_H_6_O_3_	Nist20
7	21.37	Pyrogallol	97	126.11	C_6_H_6_O_3_	Nist20
8	25.67	2-hydroxy-5-methylbenzaldehyde	86	136.15	C_8_H_8_O_2_	Nist20
9	29.29	Dihydroactinidiolide	91	180.24	C_11_H_16_O_2_	Nist20
10	36.51	Dihydroconiferyl alcohol	94	182.22	C_10_H_14_O_3_	Nist20
11	37–42	-Methyl-4-O-methyl-D-arabinopyranoside	72	178.18	C_7_H_14_O_5_	Nist20
12		-3,4, Di-O-methyl-L-arabinopyranose	72	178.18	C_7_H_14_O_5_	Nist20
13		-2-O-Methyl-D-mannopyranosa	77	194.18	C_7_H_14_O_6_	Nist20
14		-3-O-methyl-D-fructose	77	194.18	C_7_H_14_O_6_	Nist20
15		-Alpha-methyl mannofuranoside	73	194.18	C_7_H_14_O_6_	Nist20
16		-4-O-methyl-mannose	70	194.18	C_7_H_14_O_6_	Nist20
17		-4,6-di-O-methyl-alpha-d-galactose	75	208.21	C_8_H_16_O_6_	Nist20
18	43–46	3,4,6-tri-O-methyl-D-glucose	77	222.24	C_9_H_18_O_6_	Nist20
19		Methyl(methyl-4-O-methyl-alpha-D-mannopyranoside)uronate	73	236.22	C_9_H_16_O_7_	Nist20
20		Neophytadiene	97	278.5	C_20_H_38_	Nist20
21	58.83	Methyl Palmitate	99	270.5	C_17_H_34_O_2_	Nist20
22	62.31	Palmitic acid	98	256.42	C_16_H_32_O_2_	Nist20

MW: Molecular Weight, P%: Probability Percentage, MF: Molecular Formula, L: Library.

## Data Availability

The original contributions presented in the study are included in the article, further inquiries can be directed to the corresponding author.
